# Training Medical Novices in Spinal Microsurgery: Does the Modality Matter? A Pilot Study Comparing Traditional Microscopic Surgery and a Novel Robotic Optoelectronic Visualization Tool

**DOI:** 10.7759/cureus.469

**Published:** 2016-01-27

**Authors:** Marc Moisi, R. Shane Tubbs, Jeni Page, Alexandra Chapman, Brittni Burgess, Tyler Laws, Haylie Warren, Rod J Oskouian

**Affiliations:** 1 Neurosurgery, Swedish Neuroscience Institute; 2 Neurosurgery, Seattle Science Foundation; 3 Seattle Science Foundation

**Keywords:** operative microscope, surgical training, servo system, robotically controlled optoelectronic system

## Abstract

The operative microscope has been a staple instrument in the neurosurgical operating room over the last 50 years. With advances in optoelectronics, options such as robotically controlled high magnification have become available. Such robotically controlled optoelectronic systems may offer new opportunities in surgical technique and teaching. However, traditionally trained surgeons may find it hard to accept newer technologies due to an inherent bias emerging from their previous background. We, therefore, studied how a medically naïve population in a pilot study would meet set microsurgical goals in a cadaver experiment using either a conventional operative microscope or BrightMatter™ Servo system, ​a robotically controlled optoelectronic system (Synaptive Medical, Toronto, Ontario, Canada). We found that the relative ease in teaching medical novices with a robotically controlled optoelectronic system was more valuable when compared to using a modern-day surgical microscope.

## Introduction

The introduction of the operative microscope provided 3D visualization of the surgical field through magnified and illuminated binocular visualization, which dramatically changed the face of modern surgery in many subspecialties. Theodor Kurze is credited as the first neurosurgeon to perform an in vivo procedure with the operative microscope in 1957 [1]. However, with major technological advances came great strides in the field of optics. The modern day microscope has greatly changed since Dr. Kurze first utilized it, but alternate visualization platforms have also emerged. For example, a robotically controlled ultra-high magnification device, BrightMatter™ Servo system, (Synaptive Medical, Toronto, Ontario, Canada) is an example of a hands-free surgical visualization aid that could serve as an alternative to the conventional surgical microscope. This device has an optical positioning arm with an ultra-high definition camera and illumination source, which moves hands-free within the surgical suite controlled by a directional aiming device and a surgeon-controlled foot pedal. Such a device theoretically offers a relatively unobstructed surgical field and unusual surgical visualization trajectory angles not amenable to conventional surgical microscopy. This technology could also open new avenues of surgeon training as it offers identical surgical field visualization to all members of the surgical team as viewed on a large high-definition monitor. This new visualization technique is in contrast to traditional binocular microscopy, which has a limitation of two primary surgeons and camera-assisted viewing of the field.

In evaluating alternate surgical technologies, our investigators were concerned about potential bias by qualified surgeons who had been trained with surgical microscopes and had come to accept this technology as the gold standard. In order to examine the comparative ease and the learning curve of traditional surgical microscopy compared to a novel optoelectronic robotic camera system, we devised a cadaver study using medical novices who were inexperienced with either technology. We hypothesized that for medical novices the optical system they would use would make no difference in performing their surgical task. We further hypothesized that these test subjects would consider their teaching experience as more straightforward using the joint open optoelectronic robotic system compared to conventional surgical microscopy.

## Materials and methods

**Figure 1:**
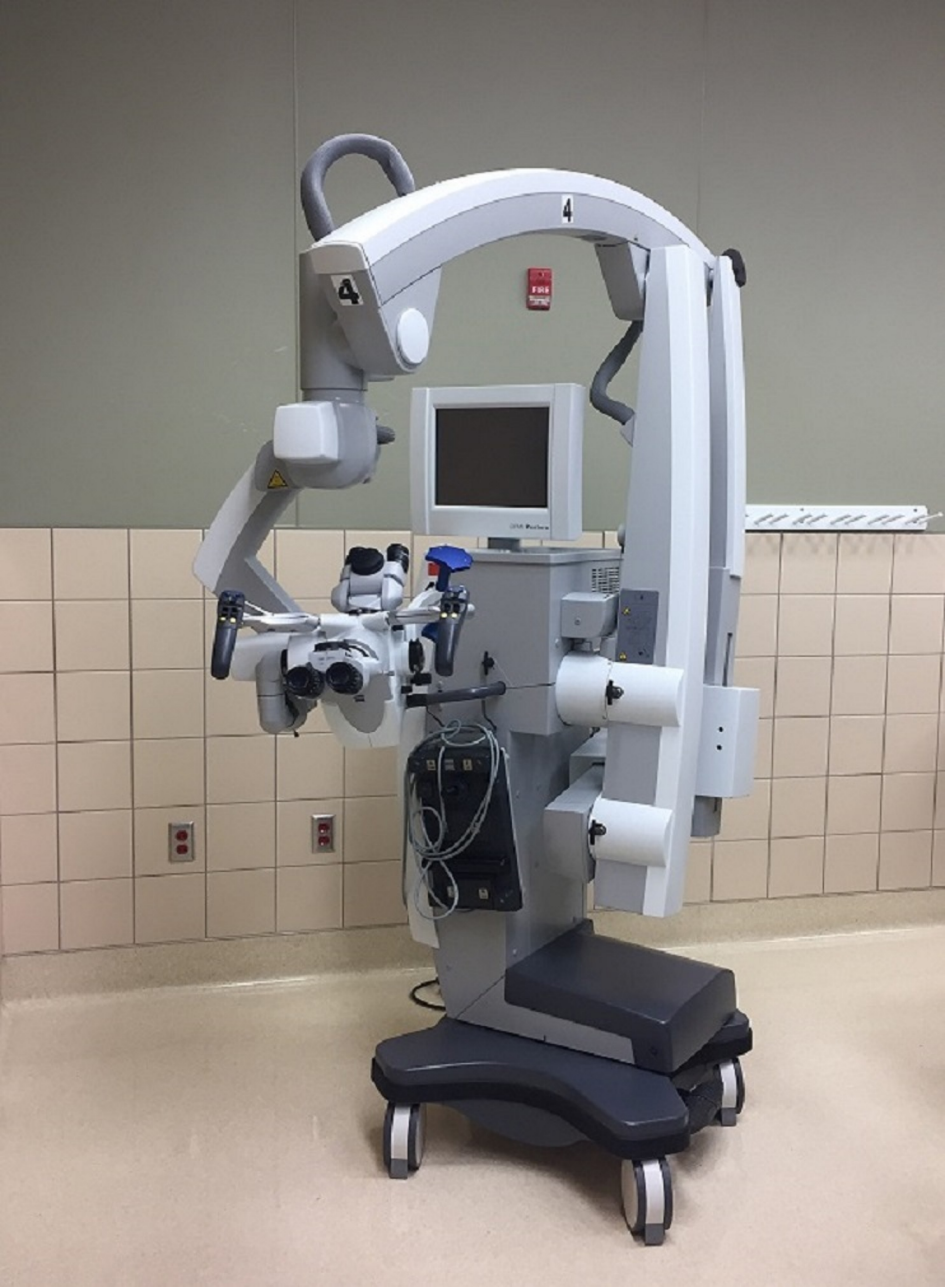
Zeiss Pentero Operative Microscope

**Figure 2:**
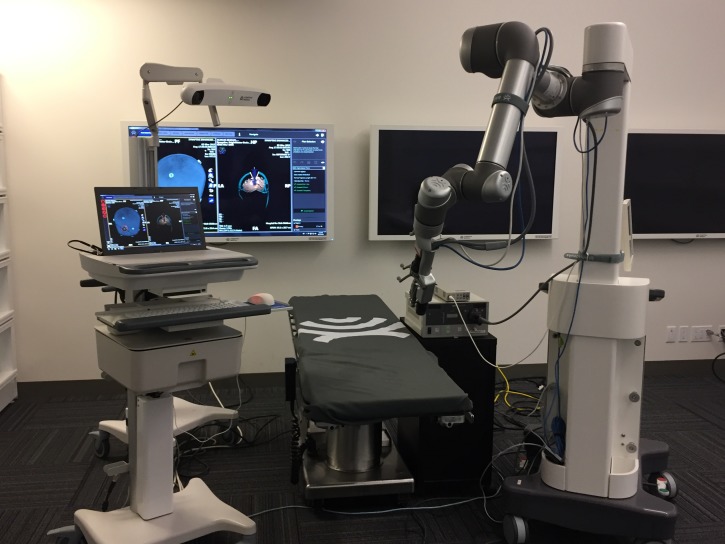
BrightMatter™ Servo system Servo system with all its components

Four medically naïve volunteers (three females and one male, aged 19-30 years) were instructed individually by an experienced neurosurgeon on how to perform a small laminotomy at the thoracolumbar junction using a consistent conventional teaching setup with anatomic aids and with typical surgical instruments, such as a high-speed burr and neurosurgical dissection tools. They then received instructions in how to use an operating microscope (Figure 1) and an optoelectronic robotic system, BrightMatter™ Servo system (Figure 2).

No participant had ever performed any form of surgical procedure since high school biology classes, and none had previously used the operative microscope or an optoelectronic robotic visualization system. The quality of the laminotomy was graded as completed by a trained surgeon observer with verbal guidance provided. The test subjects were told that the procedure was not timed. Each individual was further instructed to complete the requested procedure, to the best of their ability, using either surgical visualization technology. After completing the task with both optical systems, each participant was questioned to determine which system they felt most comfortable with, which was easier to use, and which system was better for teaching. Additional comments were documented as well.  

## Results

All four test subjects completed their surgical tasks safely and to the proctoring surgeon’s satisfaction. Upon completion of their surgical tasks, the test subjects universally expressed being content with both surgical visualization technologies. However, we noticed an overall preference towards optoelectronic robotic visualization system. As documented in Table 1, it is noted that three of the four (75%) participants felt more comfortable using the optoelectronic robotic visualization system. When deciding which was easier to use and which was better for teaching, all four participants replied that the optoelectronic robotic visualization system was the superior system in that regard.

**Table 1:** Survey Results

	Servo	Microscope
More comfortable	75%	25%
Easier to Use	100%	0
Better for teaching	100%	0

When asked to comment on the experience, the participants commented that both systems had pros and cons. One participant felt more comfortable with the operating microscope as they felt it was easier to “look down instead of forwards”. Another commented that the “microscope was easier to use, but I felt I did a faster job using Servo”. Another participant commented that the microscope was more comfortable “because they can see three-dimensional depth, but after working a little with the robotic system, I was able to use surrounding cues to work more comfortably”. It was also commented that the robotic system was easier to position since “it is automated”.

In regards to its function as a better teaching tool, the participants comments included: “The robotic camera was better because multiple people can view the same thing at the same time”, “The robotic camera allowed for a more involved teaching scenario with the instructor able to operate with me as I worked”, and “The robotic camera was better because the (proctoring) surgeon was able to point to the exact location and help guide me to the correct location. In addition, they are able to watch my exact movements with the instruments and give me tips on angles”.

**Figure 3:**
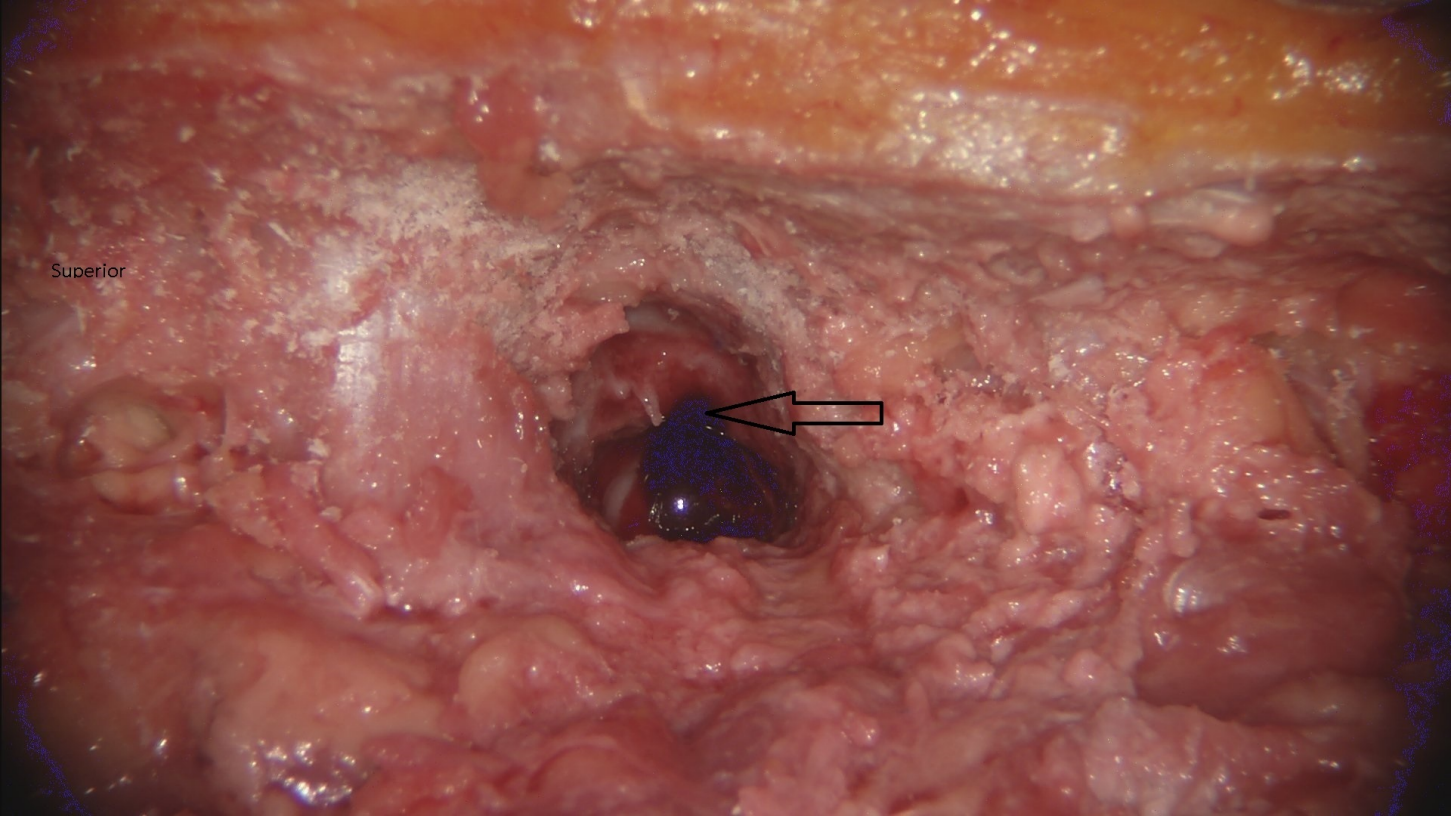
Laminotomy performed with operative microscope Left thoracic laminotomy (black arrow) was performed with the operative microscope.

**Figure 4:**
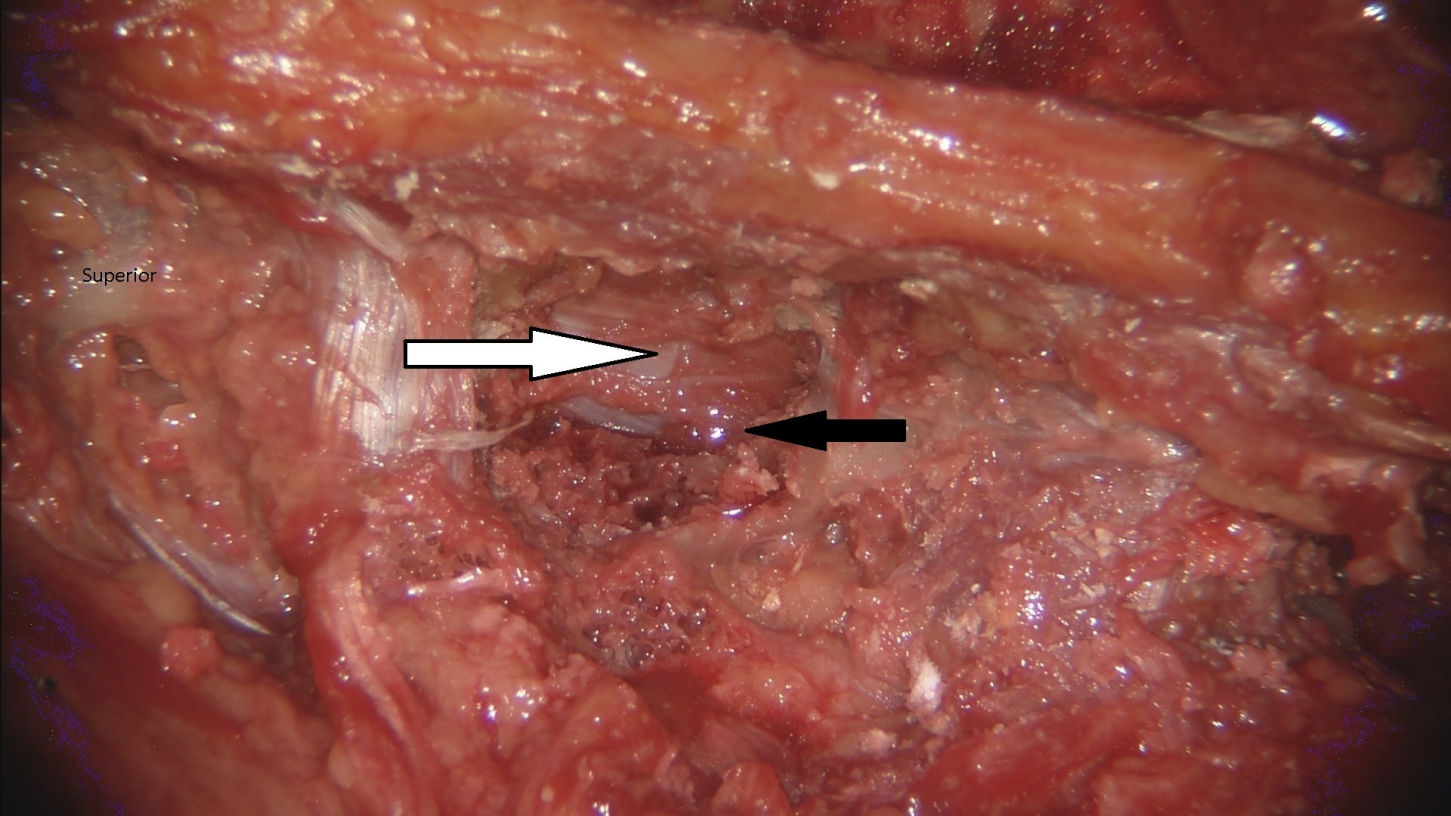
Laminotomy performed with BrightMatter™ Servo system Left thoracic laminotomy (black arrow) was performed with the BrightMatter™ Servo system. A decompression was achieved with visualization of the dura and neural structures (white arrow).

Ultimately, through judging their own work, the participants felt like they got a “cleaner” result with the optoelectric robotic system as demonstrated by the examples seen in Figures 3-4. They attributed this to the ability of being more easily guided and to the robotic camera resolution, which was described as “spectacular.”

## Discussion

The operative microscope has revolutionized the field of neurosurgery, enabling the surgeon to better visualize the targeted pathology with less invasiveness, providing the ability for an assistant to see down the same small corridor, as well as creating a modality for teaching new surgeons. All new residents are taught on the microscope, and as their career advances, each surgeon becomes increasingly proficient with its use. 

Although there have been some advances with the operative microscope, the basic technology has not substantially evolved over the last few decades. In the interim, other optical visualization platforms have emerged, such as the one tested here. The question arises as to how and under what circumstances can a new technology displace decades of experience with a proven technology? We realize that there are multiple dimensions to this question, and we suggest taking a *de novo* approach to understanding the end user perspective. This can be done b,y taking the vantage point of an unbiased medical novice, studying their observations, and drawing insightful conclusions prior to moving on to more detailed surgeon-based investigations. With this question, we investigated how medically naïve and unbiased individuals viewed such newer technology in comparison with the traditional and established technology.

Initially, three of the four participants felt more comfortable using the microscope. However, after becoming accustomed to the optical robot, all four of the novices preferred the newer technology. The reason for the initial comfort level of the microscope was its three-dimensional capabilities and the easier collinear location of anatomical landmarks, as well the “watching exactly what your hands are doing”. One participant commented that the microscope allowed them to “make more definite movements”.

The teaching capabilities offered by the visual display of the optical robot seemed to be the biggest advantage for the participants. They are able to be educated with hands-on training with a surgical proctor watching at all times. This technology also allowed for simultaneous collinear viewing by multiple surgeons in contrast to surgical microscopy with its optic limitations.

Although this unbiased group supported the use of the optoelectronic robotic visualization system, newer technologies cannot and should not be automatically implemented into the operative theater without due diligence. As with any new technique, there is concern about the possible impact of a learning curve. A suitable first practice and study environment for new technology presents itself in a cadaveric lab where the surgeon can investigate strengths and limitations of a newer technology at their own pace. Gasco, et al. found that “Cadaver simulations accrued the highest reported benefit” for teaching residents [2]. This can be extrapolated to senior physicians who can more readily bypass the learning curve, reaching competency earlier in a non-operative theater environment. In these instances, it can be assumed the physicians will be willing to more quickly implement an optoelectronic robotic visualization system, such as the one studied into their daily practices. Future studies could include a comparison between surgeons completing procedures with the microscope and with Servo with the purpose of examining the difference in the quality of the procedure or the time spent.

## Conclusions

In a small medically naïve population, the relative ease and potential for teaching with an optoelectronic robotic visualization aid was determined to be more valuable when compared to the modern day microscope. The future of the surgical field will require a tool that is both versatile in high-powered resolution as well as an optimized teaching tool for future surgeons. Further investigation, however, with more senior practitioners is still necessary before determining a true leader in this quickly advancing field.
